# Burden of leishmaniasis in Brazil and federated units, 1990-2016: Findings from Global Burden of Disease Study 2016

**DOI:** 10.1371/journal.pntd.0006697

**Published:** 2018-09-06

**Authors:** Juliana Maria Trindade Bezerra, Valdelaine Etelvina Miranda de Araújo, David Soeiro Barbosa, Francisco Rogerlândio Martins-Melo, Guilherme Loureiro Werneck, Mariângela Carneiro

**Affiliations:** 1 Epidemiology of Infectious and Parasitic Diseases Laboratory, Department of Parasitology, Institute of Biological Sciences, Universidade Federal de Minas Gerais, Belo Horizonte, Minas Gerais, Brazil; 2 Secretariat of Health Surveillance, Ministry of Health, Brasília, Distrito Federal, Brazil; 3 Federal Institute of Education, Science and Technology of Ceará, Caucaia, Ceará, Brazil; 4 Institute for Public Health Studies, Universidade Federal do Rio de Janeiro, Rio de Janeiro, Rio de Janeiro, Brazil; 5 Post-Graduation Program in Health Sciences, Infectology and Tropical Medicine, Universidade Federal de Minas Gerais, Belo Horizonte, Minas Gerais, Brazil; Institute of Tropical Medicine, BELGIUM

## Abstract

**Background:**

The study presents estimates for the burden of visceral leishmaniasis (VL) and cutaneous and mucocutaneous leishmaniasis (CML) in Brazil and its 27 federated units using data from the Global Burden of Disease Study (GBD) 2016.

**Methodology:**

We report the incidence, years of life lost (YLL), years lived with disability (YLD), and disability-adjusted life years (DALY) for leishmaniasis in Brazil from 1990 to 2016. The metrics are presented as age-standardized rates per 100,000 inhabitants with their respective uncertainty intervals (95%UI) and relative percentages of change.

**Principal findings:**

The age-standardized incidence rate of leishmaniasis decreased 48.5% from 1990 (71.0, 95%UI 24.3–150.7) to 2016 (36.5, 95%UI 24.7–50.9), whereas the age-standardized DALY increased 83.6% over the studied period from 12.2 (95%UI 7.9–18.8) to 22.4 (95%UI 13.3–36.2). The age-standardized incidence rate and YLL for VL increased by 52.9% and 108% from 1990 to 2016, respectively. Considering CML, the age-standardized incidence rate and YLD decreased by 51% and 31.8% respectively for the same period. For VL, similar profiles for male and female were observed, with YLL and DALY increasing over time; with males presenting slightly higher values. The highest YLL rates were among "under 1-year old" children, which increased 131.2% from 1990 to 2016. Regarding CML, the highest values of YLD and DALY were verified among males, and YLD values showed a similar profile, with rates increasing with age. The VL burden increased in some states in the Northeast and Southeast regions and decreased for CML in some Northern states.

**Conclusion:**

The increase of VL burden over the study period might be associated with the difficulties in controlling the disease spread. Information regarding the weight of VL and CML, including the death and disability tolls that they cause, highlights the impact of these neglected diseases on public health and the importance of effective prevention and treatment.

## Author summary

Leishmaniasis are diseases caused by obligatory intracellular parasites of the genus *Leishmania* and are transmitted to humans through the bite of female sandflies during blood repast. Untreated visceral leishmaniasis can lead to death, while cutaneous and mucocutaneous forms generally do not pose risk of death but can cause disability and permanent injury, which raises stigma and social prejudice. The Global Burden of Disease Study (GBD) is a systematic and scientific effort to quantify the health loss caused by infectious and non-infectious diseases and injury and their risk factors categorized by age, sex, and geographic distribution at specific periods of time. The present article describes, for the first time, the burden of leishmaniasis in the 27 Brazilian federated units. The VL burden increased in some states in the Northeast and Southeast regions and decreased for CML in some Northern states. Understanding the burden of these diseases and their regional differences is of great relevance for the establishment of adequate and region-specific surveillance and control measures. In addition, it can help in the rational use of available resources and in decision making aimed at reducing the transmission of the parasite and the burden of this disabling and potentially lethal disease.

## Introduction

Leishmaniasis are neglected tropical diseases (NTD) caused by protozoan parasites of the genus *Leishmania* [1,2]. The two distinct clinical forms of the disease are the visceral leishmaniasis (VL) and the tegumentary leishmaniasis, which comprises cutaneous leishmaniasis, mucocutaneous leishmaniasis, and diffuse cutaneous leishmaniasis [3,4]. The World Health Organization (WHO) estimates the annual global occurrence of 400,000 new cases of VL and 1 million new cases of cutaneous and mucocutaneous leishmaniasis (CML) [2,5].

In the Americas, VL is present in 12 countries [6], with 96% of the cases being reported in Brazil (4,200 to 6,300 cases per year) [1] while CML occurs in 20 American countries, being endemic in 18 of them, albeit with different transmission intensities [6]. In Brazil, CML affects 72,800 to 119,600 people annually [1]. From1990 to 2016, 84,922 cases of VL were confirmed in Brazil, with the case-fatality rate reaching 7.4% in 2016 [7]. In the same period, 687,780 cases of CML have been reported, albeit with low mortality [8].

Identifying diseases that pose the greatest threat to health and wellbeing helps policy-makers planning interventions, monitoring processes, and evaluating the impact and effectiveness of control measures. The burden of a disease reflects the human and economic costs caused by the disease and, better than morbidity and mortality indicators, reflects the relative importance of the disease and disability for the entire population [9,10]. Although the burden of leishmaniasis in Brazil has been quantified at a national level, disparities are observed among different regions [11,12], and subnational analyses are still missing.

The Global Burden of Disease (GBD) is a descriptive epidemiologic study that, since 1990, quantifies and compares the magnitude of health loss due to diseases and injuries and the risk factors associated with location, gender, age, and time. The GBD study uses the disability-adjusted life year (DALY), a measure of health loss due to both fatal and non-fatal disease burden, as the main population health metric. DALYs are estimated by summing years lived with disability (YLDs) and years of life lost (YLLs) due to premature mortality for a given cause [11,12].

The 2013 GBD estimated a total of 25.17 million DALYs for the 17 NTDs prioritized by the World Health Organization and other NTDs in that year; of these, 4.24 million were attributed to VL and 40,000 DALYs were attributed to CML [13]. Despite the availability of the GBD raw data, detailed regional analyses are still needed as they could help designing more effective strategies for controlling the diseases.

Although the burden of leishmaniasis in Brazil has been quantified at a national level, clear disparities are observed among different regions [11,12] and subnational analyses are still missing. Identifying levels and trends of leishmaniasis’ burden may help health authorities plan interventions, monitor processes, and evaluate the impact and effectiveness of the adopted disease control measures [9,10]. Herein we analyzed the burden of VL and CML using data from the GBD 2016 for Brazil and its 27 federated units. To the best of our knowledge, this is the first nation-wide analysis of VL and CML burden stratified by gender, age group, and federated units in Brazil, one of the countries most affected by leishmaniasis in the world.

## Methods

### Ethical considerations

The protocol for this study was approved by the Research Ethics Committee of the Federal University of Minas Gerais (Project CAAE 62803316.7.0000.5149).

### Study area

The Federative Republic of Brazil is the largest country in Latin American and the fifth largest in the world in territory by area, with 8,515,759,090 Km^2^ (equivalent to 47% of the South American territory). Regarding population size, the country is the sixth largest, with 207.7 million inhabitants in 2017 [14].

Brazil is politically and administratively divided into 27 federated units (26 states and the Federal District) and 5,570 municipalities. The 27 federated units are grouped into five geographic regions: Central-West, Northeast, North, Southeast, and South [14].

### GBD overview and case definition

The GBD is a project developed by the Institute for Health Metrics and Evaluation with the aim of creating a comprehensive and up-to-date roadmap of the health problems worldwide [15]. The general methodological approaches used by GBD 2016 to estimate the metrics are detailed in previous publications [11,12].

The GBD 2016 provides a comprehensive annual assessment of mortality and morbidity estimates for 333 diseases and injuries and 84 risk factors for 195 countries and territories from 1990 to 2016 [16, 17]. The GBD 2016 cause list hierarchy is organized in four levels of causes that are mutually exclusive and collectively exhaustive [16,17]. Leishmaniasis are within the level 2 category “NTDs and malaria”, which consists of 20 infectious and parasitic diseases including malaria, NTDs prioritized by the WHO, and other important neglected diseases such as yellow fever and Ebola virus disease. Among the parasitic diseases, leishmaniasis are divided into VL and CML [16,17]. In the GBD 2016, NTD causes were defined and identified according to the International Classification of Diseases, 9^th^ (ICD-9) and 10^th^ (ICD-10) Revisions. The specific ICD definitions and modeling strategy for the cause of each leishmaniasis are described in detail elsewhere [16,17].

### Data sources and analysis

The present study describes the metrics generated by the GBD 2016 on leishmaniasis (VL and CML) in Brazil and its 27 federated units according to time, gender, and age. GBD data sources for Brazil have been described elsewhere [18,19,20]. The GBD mortality data, as well as the generation for the YLL estimates, in Brazil come from the Brazilian Mortality Information System (Sistema de Informação sobre Mortalidade—SIM) adjusted by other national and international sources. The main sources of morbidity data, such as the estimates for YLD, are the Information System of Diseases Notification (Sistema de Informação de Agravos de Notificação—SINAN), the Hospital Information System of the Unified Health System (Sistema de Informações Hospitalares do Sistema Único de Saúde—SIH/SUS) and the Outpatient Information System of the Unified Health System (Sistema de Informações Ambulatoriais do Sistema Único de Saúde—SIA/SUS). Additional estimates published in scientific literature on the prevalence of diseases from Brazilian population-based studies and databases from leishmaniasis control programs were used [18,19,20].

For the GBD, each death is attributed to a single underlying cause—the cause that initiated the series of events leading to death, in accordance with the ICD principles [16]. In the GBD 2016, data corrections were made for mortality sub-registration and redistribution of garbage codes for defined causes based on the GBD 2016 redistribution algorithms [16]. Garbage codes are the assignment of causes of death that could not or should not be classified as the underlying cause of death [16]. GBD 2016 used Cause of Death Ensemble model (CODEm), negative binomial regression, and natural history models to estimate the number of deaths for NTD causes by location, age, gender, and year. For CML, the GBD study assumed that mortality was null over the years and, therefore, the YLD values were the same as for DALY, which was also described in a previous study [22]. These modeling strategies for estimating fatal VL were described in detail elsewhere [16].

The modeling strategy for morbidity estimation and validation of the GBD 2016 has been published elsewhere [17]. The GBD study uses all available data that meet a minimum standard of acceptable quality for each disease. GBD 2016 used DisMod-MR 2.1, a Bayesian-regression analytic tool, to synthesize consistent estimates of prevalence and incidence of non-fatal outcomes by age, gender, year, and location using a wide range of updated and standardized analytical procedures [17].

The methods used by GBD 2016 to estimate the metrics are detailed in previous publications [11,12]. The leishmaniasis burden were assessed by the following metrics: incidence; years of life lost due to premature death (YLL); years lived with disability (YLD); and disability-adjusted life year (DALY = YLL + YLD). YLL expresses the effect of premature deaths on the population and results from the multiplication of the number of deaths due to leishmaniasis in each age group by the standard life expectancy at age group. For GBD 2016, the standard life expectancy at birth is 86.6 years, based on the lowest observed death rates for each 5-year age group in populations greater than 5 million people in 2016 [21]. YLD expresses the sum of the prevalence of sequelae related to leishmaniasis multiplied by a disability weight [11,12]. Disability weight reflects the severity of health loss associated with the respective disease and it is presented on a scale varying from 0 (perfect health) to 1 (equivalent to death) [21]. The sum of YLL and YLD yields DALY [11,12]. The estimates are shown as age-standardized rates by 100,000 inhabitants.

We ranked the federated units from the highest to the lowest value of YLL for VL and from the highest to the lowest value of YLD for CML. Age-standardized rates were calculated using GBD world population standard. The metrics were presented with their respective 95% uncertainty intervals (95%UIs) and the relative percentages of change.

## Results

**Fig 1:**
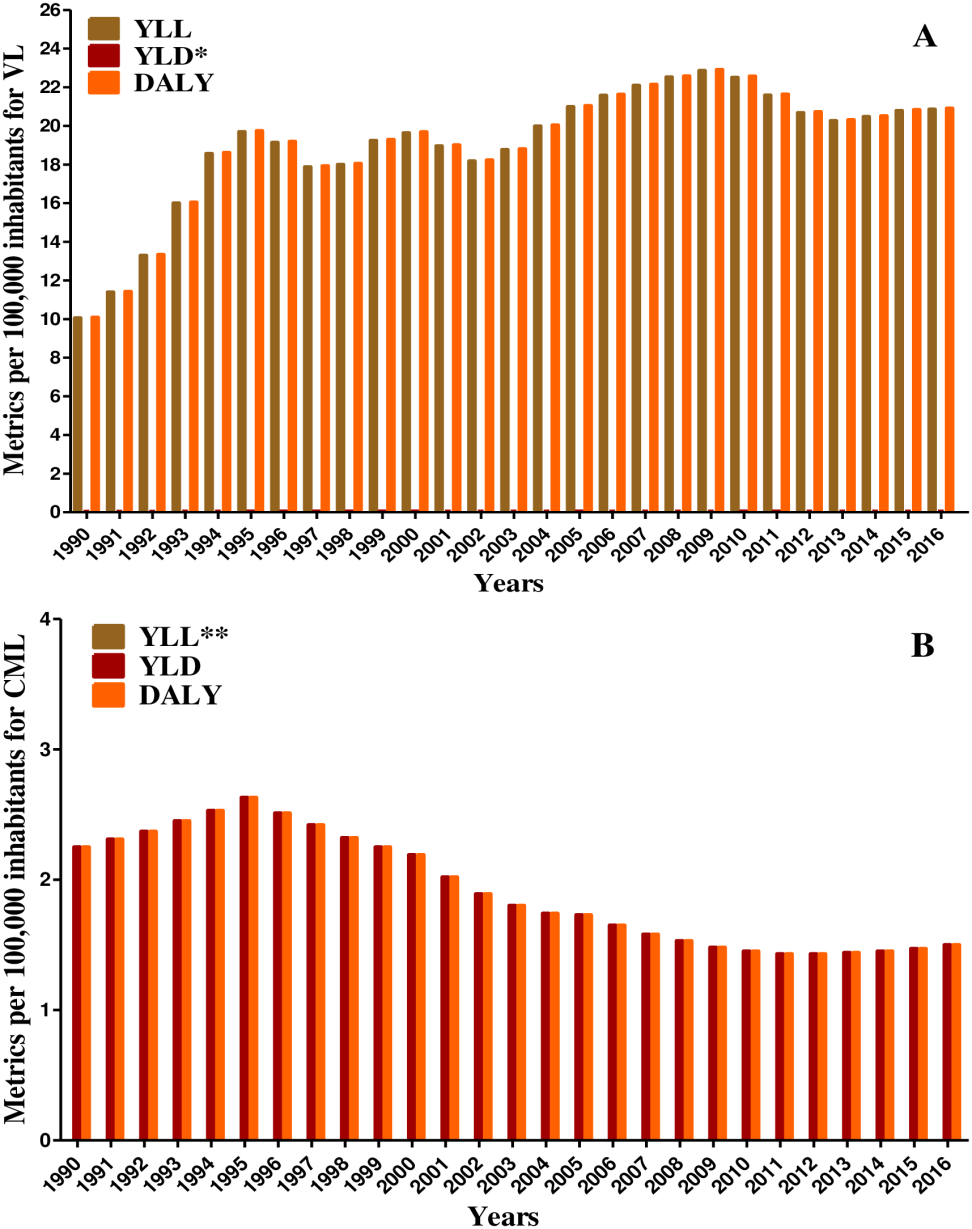
Age-standardized rates of years of life lost (YLL), years lived with disability (YLD), and disability-adjusted life years (DALY) per 100,000 inhabitants for Visceral Leishmaniasis (VL) (A) and Cutaneous and Mucocutaneous Leishmaniasis (CML) (B) per 100,000 inhabitants, Brazil, 1990 to 2016, GBD Study 2016. *Visceral Leishmaniasis values of YLD are less than one. **Cutaneous and Mucocutaneous Leishmaniasis mortality was assumed to be zero and for this reason YLL values are zero.

The main metrics on the burden of leishmaniasis and the percentage variations between 1990 and 2016 in Brazil are presented in Table 1. Noteworthy, the incidence rates of leishmaniasis decreased 48.5% from 1990 to 2016, whereas the DALY increased 83.6% over the same period; the increase of DALY was mainly due to the expressive increase of YLL (108%) (Table 1 and Fig 1A and 1B).

**Table 1:** Age-standardized rates of incidence and mortality for leishmaniasis per 100,000 inhabitants and relative change regarding years of life lost (YLL), years lived with disability (YLD), and disability-adjusted life years (DALY), Brazil,1990–2016, GBD Study 2016.

Metrics	Rate per 100,000 (95%UI)			Relative change (%)		
	1990	2000	2016	1990 X 2000	2000 X 2016	1990 X 2016
**Leishmaniasis**						
**Incidence**	71.0 (24.3–150.7)	67.2 (32.4–124.5)	36.5 (24.7–50.9)	-5.3	-45.6	-48.5
**YLL**	10.0 (6.0–16.3)	19.6 (11.3–32.1)	20.9 (11.7–34.7)	96.0	6.1	108
**YLD**	2.2 (0.7–5.0)	2.2 (1.0–4.4)	1.5 (0.9–2.3)	0	-31.8	-31.8
**DALY**	12.2 (7.9–18.8)	21.7 (13.3–34.2)	22.4 (13.3–36.2)	77.8	3.2	83.6
**Visceral Leishmaniasis**						
**Incidence**	1.7 (1.4–2.1)	2.9 (2.7–3.2)	2.6 (2.5–2.8)	70.5	-10.3	52.9
**YLL**	10.0 (6.0–16.3)	19.6 (11.3–32.1)	20.9 (11.7–34.7)	96.0	6.1	108
**YLD**	0.03 (0.02–0.04)	0.05 (0.03–0.07)	0.04 (0.03–0.06)	67.7	-9.6	51.6
**DALY**	10.0 (6.1–16.4)	19.6 (11.4–32.1)	20.9 (11.8–34.8)	96.0	6.6	109
**Cutaneous and Mucocutaneous Leishmaniasis**						
**Incidence**	69.3 (22.4–148.9)	64.3 (29.6–121.6)	33.9 (22.0–48.3)	-7.2	-47.2	-51.0
	0.0	0.0	0.0	0.0	0.0	0.0
YLL*	0.0	0.0	0.0	0.0	0.0	0.0
**YLD**	2.2 (0.6–4.9)	2.1 (0.9–4.3)	1.5 (0.9–2.3)	-4.5	-31.5	-31.8
**DALY**	2.2 (0.6–4.9)	2.1 (0.9–4.3)	1.5 (0.9–2.3)	-4.5	-31.5	-31.8

*Cutaneous and Mucocutaneous Leishmaniasis mortality was assumed to be zero; 95%UI: 95% uncertainty interval.

The metric increased for VL and decreased for CML over the years. Considering VL, the age-standardized incidence rate per 100,000 inhabitants and the YLL increased 52.9% and 108% from 1990 to 2016, respectively. On the other hand, CML showed a 51% decrease in the age-standardized incidence rate per 100,000 inhabitants and a 31.8% decrease of the YLD (Table 1).

**Fig 2:**
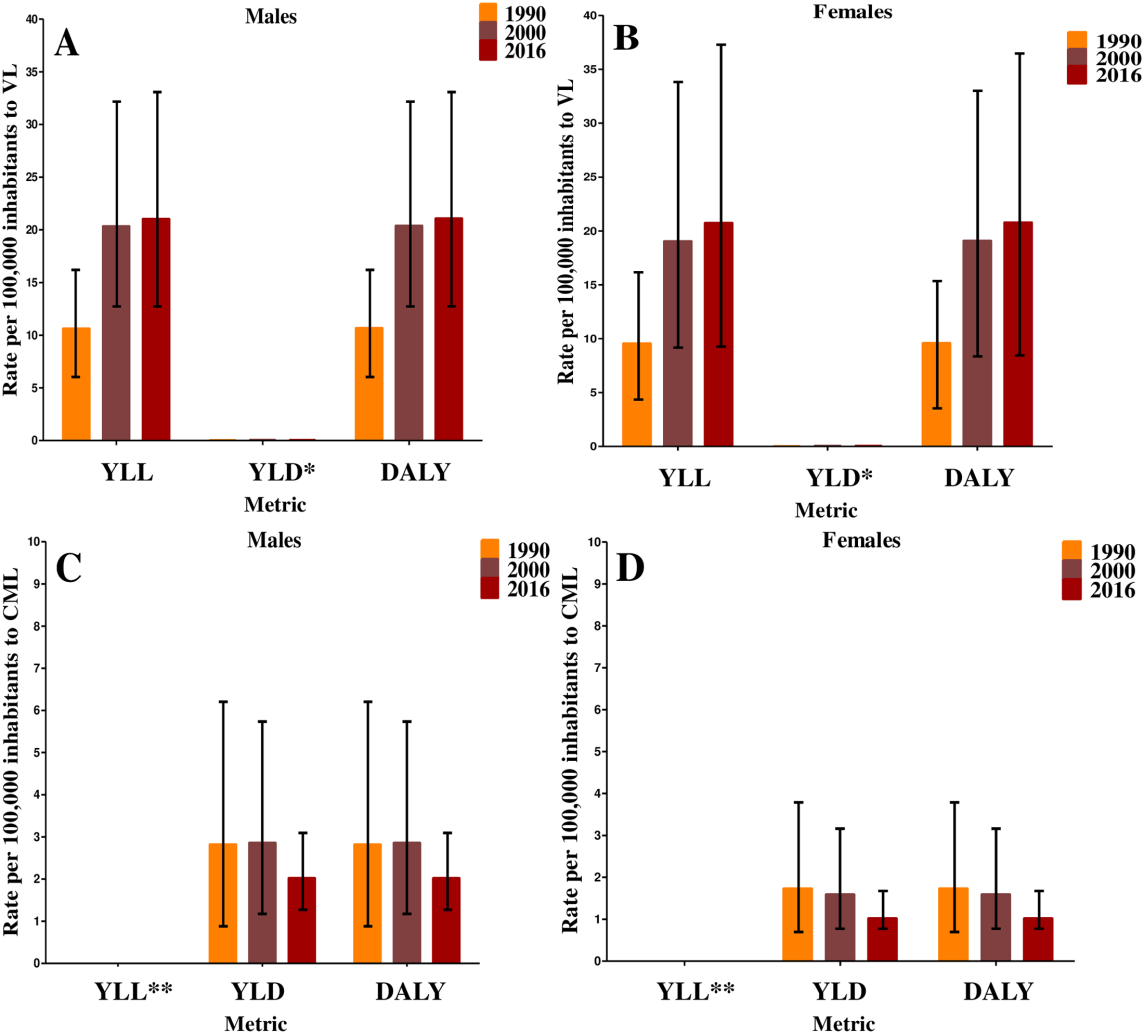
Age-standardized rates of years of life lost (YLL), years lived with disability (YLD), and disability-adjusted life years (DALY) per 100,000 inhabitants for Visceral Leishmaniasis (VL) and Cutaneous and Mucocutaneous Leishmaniasis (CML) in 1990, 2000, and 2016, by gender. (A) Rates per 100,000 inhabitants for males with VL. (B) Rates per 100,000 inhabitants for females with VL. (C) Rates per 100,000 inhabitants for males with CML. (D) Rates per 100,000 inhabitants for females with CML. *Visceral Leishmaniasis values of YLD are less than one. **Cutaneous and Mucocutaneous Leishmaniasis mortality was assumed to be zero and for this reason YLL values are zero. GBD Study 2016.

When assessing the GBD indicators of VL by gender, we observed similar profiles for both genders, with low values of YLD, and YLL and DALY increasing over time for males and females. Males presented slightly higher values for all these metrics in comparison with females (Fig 2A and 2B). Regarding CML, the highest values of DALY were verified among males (Fig 2C and 2D).

**Fig 3:**
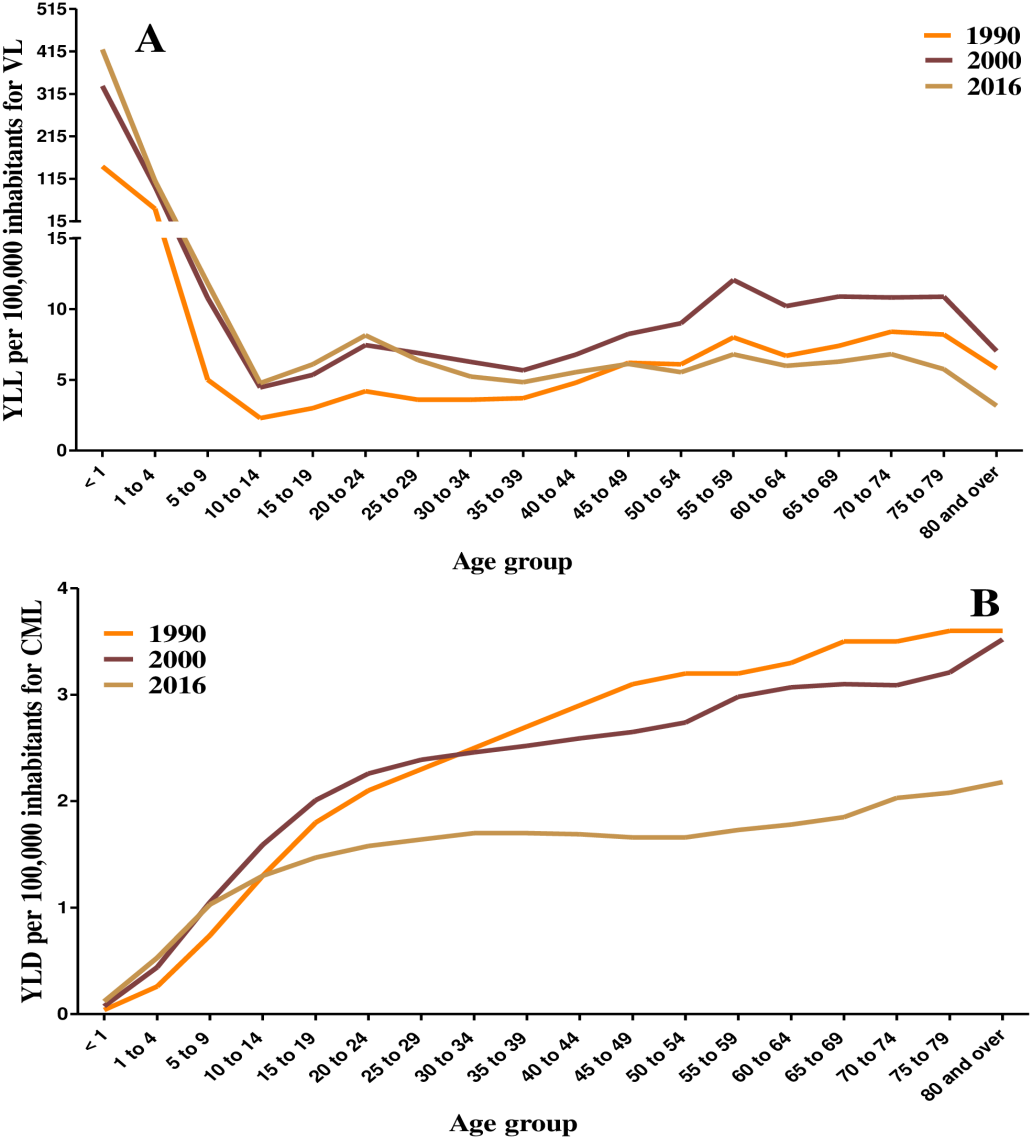
Age-standardized rates of years of life lost (YLL) for Visceral Leishmaniasis (VL) (A) and years lived with disability (YLD) for Cutaneous and Mucocutaneous Leishmaniasis (CML) (B) per 100,000 inhabitants by age group, Brazil in 1990, 2000 and 2016. CML mortality was assumed to be zero; YLD is low for VL (premature mortality predominates in VL). GBD Study 2016.

Given that the primary contributor for the burden of the DALY corresponds to YLL for VL and YLD for CML, the analysis by age and federated units were performed using the values of YLL for VL and YLD for CML. Fig 3 shows the YLL for VL and the YLD for CML by age. The highest rates of VL were observed among "under 1-year old" children: 144.3 (95%UI 60.6–255.9) in 1990, 333.7 (95%UI 158.1–603.8) in 2000, and 419.5 (95%UI 207.1–770.6) in 2016. The YLL for VL among children at this age increased by 131.2% between the years 1990 and 2000 and by 25.7% between 2000 and 2016. The second highest YLL for VL corresponded to “1 to 4 years old” children, while the YLL values in the other age groups were very low in the years evaluated (Fig 3A). For CML, YLD values showed a similar profile in the years 1990, 2000, and 2016, with disability rate increasing with age. The YLD decreased over the period for all age groups (Fig 3B).

**Fig 4:**
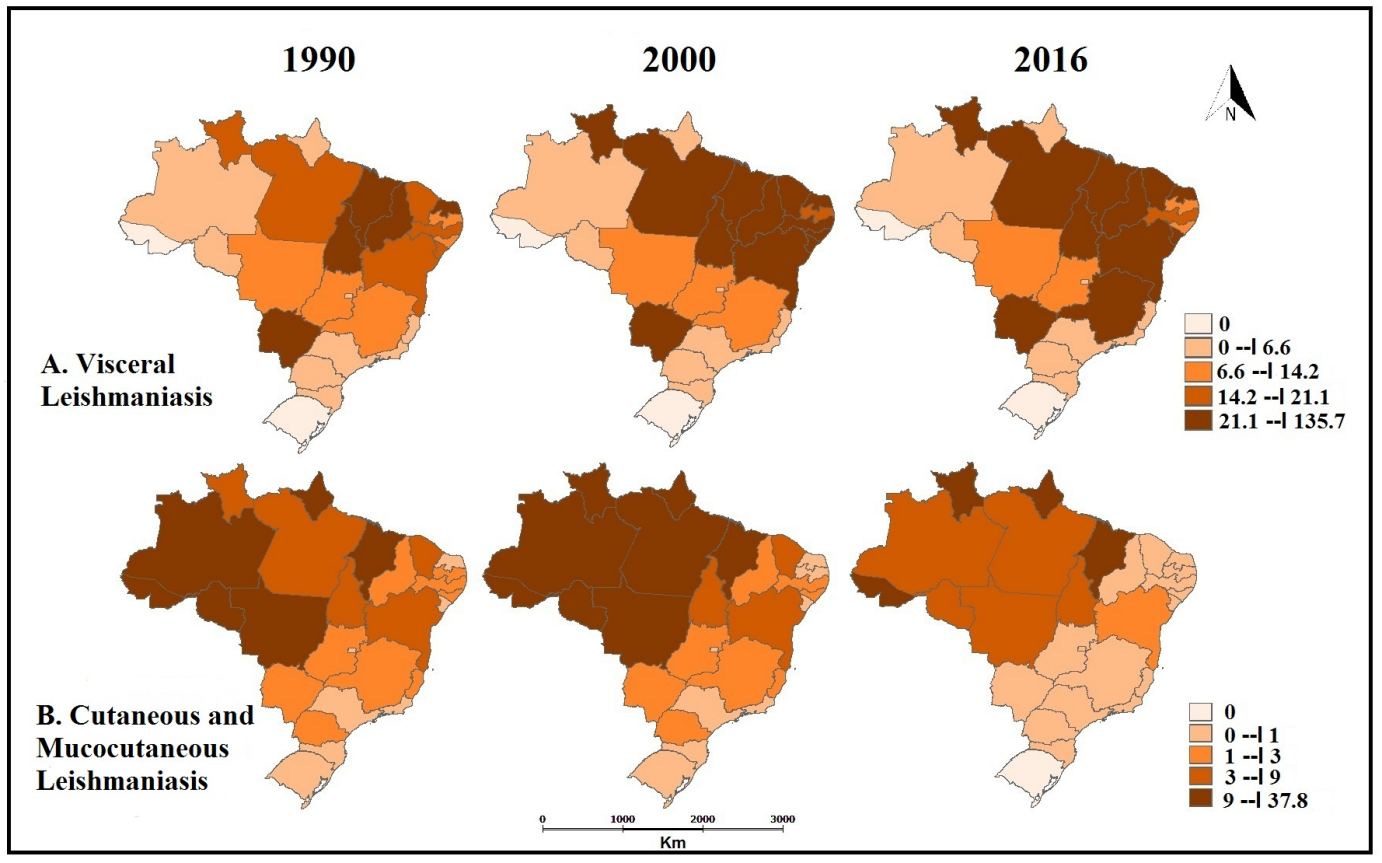
Age-standardized rates of years of life lost (YLL) for Visceral Leishmaniasis (A) and years lived with disability (YLD) for Cutaneous and Mucocutaneous Leishmaniasis (B) per 100,000 inhabitants in 1990, 2000 and 2016 considering the federated units of Brazil. GBD Study 2016.

**Fig 5:**
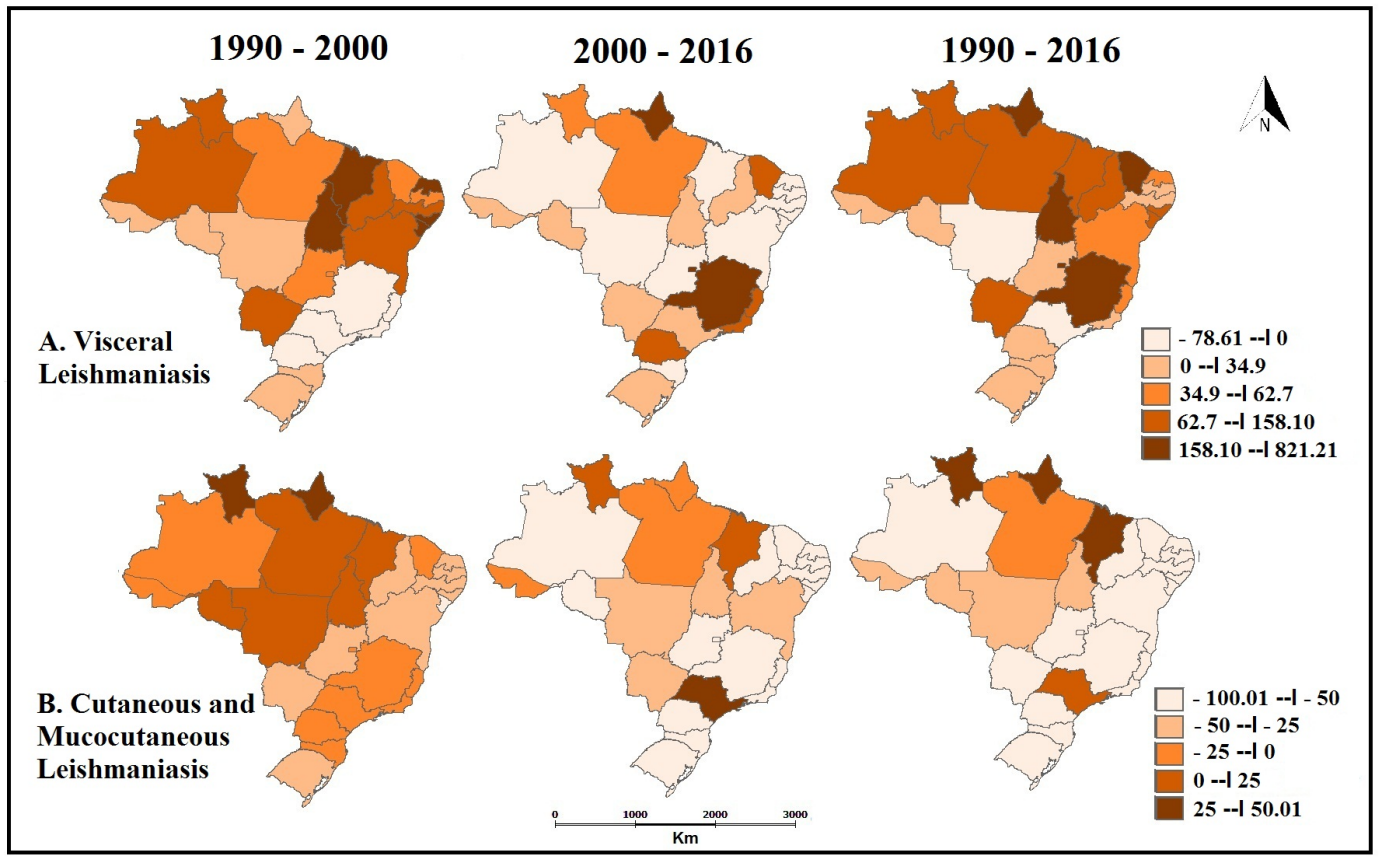
Percentages of change calculated for years of life lost (YLL) for Visceral Leishmaniasis (A) and years of life lost (YLD) for Cutaneous and Mucocutaneous Leishmaniasis (B) per 100,000 inhabitants from 1990to 2016 considering Brazil’s federated units. GBD Study 2016.

The estimated values of YLL for VL per 100,000 inhabitants and the ranking of Brazilian states in the years 1990, 2000, and 2016 are shown in Table 2 and Fig 4A. The state of Maranhão (Northeast Region) had the highest values of YLL in 1990 (41.2; 95%UI 23.6–69.8) and in 2000 (111.7; 95%UI 61.0–188.9). In 2016, the highest value of this metric was observed in the state of Tocantins (North Region) (135.7; 95%UI 77.8–221.5). The YLL increased from 1990 to 2000 and from 2000 to 2016 in the states of Tocantins, Pará and Roraima (North Region), Piauí and Ceará (Northeast Region), and Mato Grosso do Sul (Central-West Region) (Figs 4A and 5A).

**Table 2:** Age-standardized rates of years of life lost (YLL) per 100,000 inhabitants for Visceral Leishmaniasis considering the Brazilian federated units between 1990 and 2016, GBD Study 2016.

Federated units	Year					
	1990		2000		2016	
	YLL (95%UI)	Ranking	YLL (95%UI)	Ranking	YLL (95%UI)	Ranking
**Maranhão**	41.2 (23.6–69.8)	1*	111.7 (61.0–188.9)	1*	92.9 (49.8–164.2)	2*
Tocantins**	40.3 (23.2–62.9)	2*	108.5 (64.1–175.2)	2*	135.7 (77.8–221.5)	1*
Piauí**	33.9 (20.2–53.7)	3*	66.5 (37.3–111.4)	4	79.7 (44.5–135.1)	3*
Mato Grosso do Sul**	21.7 (12.7–33.3)	4	41.3 (23.9–66.3)	7	50.5 (29.0–80.9)	5
**Rio Grande do Norte**	21.1 (12.2–33.2)	5	67.1 (38.7–111.9)	3*	29.2 (14.9–51.9)	9
Pará**	19.6 (12–30.3)	6	26.9 (15.9–44.1)	11	39.3 (21.5–68.3)	6
Ceará**	19.2 (11.6–31.7)	7	25.9 (14.8–42.5)	12	57.3 (30.3–100.3)	4
**Bahia**	18.9 (11.0–31.7)	8	33.3 (18.6–55.8)	8	26.3 (13.9–46.3)	10
**Sergipe**	17.2 (10.0–26.7)	9	44.4 (25–72.8)	6	31.8 (14.8–55.3)	9
Roraima**	16.6 (7.0–29.9)	10	27.0 (12.1–44.4)	10	39.0 (16.0–65.9)	7
**Pernambuco**	14.2 (8.8–22.8)	11	32.0 (17.8–54)	9	15.1 (8.3–26.4)	11
**Paraíba**	12.4 (6.9–19.5)	12	19.9 (10.3–32)	13	13.7 (5.8–24.7)	12
**Minas Gerais**	10.1 (6.1–16.9)	13	9.5 (5.3–16.1)	15	32.6 (17.2–58.7)	8
**Mato Grosso**	8.1 (4.1–13.4)	14	8.1 (3.6–13.7)	16	7.1 (2.8–12.7)	15
**Goiás**	6.9 (3.7–10.9)	15	9.9 (5–15.9)	14	8.0 (3.6–14.1)	14
**Alagoas**	6.6 (3.5–11.1)	16	60.8 (35.6–101)	5	13.0 (5.6–23.6)	13
**São Paulo**	5.8 (3.4–9.5)	17	4.4 (2.4–7.3)	17	5.7 (3.1–9.7)	16
**Espírito Santo**	2.1 (0.9–3.8)	18	1.7 (0.6–3.4)	18	2.9 (0.7–5.8)	18
**Distrito Federal**	0.8 (0.3–1.4)	19	1.3 (0.5–2.5)	19	3.6 (0.9–7)	17
**Paraná**	0.7 (0.3–1.2)	20	0.4 (0.1–0.8)	22	0.9 (0.2–1.9)	19
**Rondônia**	0.7 (0.3–1.1)	20	0.7 (0.2–1.2)	20	0.7 (0.2–1.4)	21
**Rio de Janeiro**	0.6 (0.2–1)	21	0.4 (0.1–0.8)	22	0.7 (0.2–1.4)	21
**Amapá**	0.3 (0.2–0.6)	22	0.3 (0.1–0.5)	23	0.8(0.3–1.6)	20
**Amazonas**	0.3 (0.1–0.5)	22	0.6 (0.3–1.1)	21	0.5 (0.2–0.9)	22
**Santa Catarina**	0.3 (0.1–0.6)	22	0.4 (0.1–0.7)	22	0.3 (0.1–0.7)	23
**Acre**	0.0 (0.0–0.0)	23	0.0 (0.0–0.0)	24	0.0 (0.0–0.0)	24
**Rio Grande do Sul**	0.0 (0.0–0.0)	23	0.0 (0.0–0.0)	24	0.0 (0.0–0.0)	24

YLL: years of life lost; 95%UI: 95% uncertainty interval of;

* The three highest values of YLL according to the ranking of states per year;

** States with increase in YLL values between 1990 to 2000 and 2000 to 2016 comparisons, according to percentage change calculation. Premature mortality predominates in VL.

The estimated values of YLD for CML per 100,000 inhabitants and the ranking of Brazilian states in the years 1990, 2000, and 2016 are presented in Table 3 and Fig 4B. The state of Acre (North Region) showed the highest rates in the three years evaluated: 37.8 (95%UI 0.0–193.9) in 1990, 35.2 (95%UI 2.3–126.0) in 2000, and 27.0 (95%UI 8.1–67.0) in 2016. YLD decreased in most Brazilian states, with the exception of Maranhão (Northeast Region) and Roraima (North Region) (Figs 4B and 5B).

**Table 3:** Age-standardized rates of years lived with disability (YLD), per 100,000 inhabitants, for Cutaneous and Mucocutaneous Leishmaniasis and relative change in the Brazilian federated units between 1990 and 2016, GBD Study 2016.

Federated Units	Year					
	1990		2000		2016	
	YLD (95%UI)	Ranking	YLD (95%UI)	Ranking	YLD (95%UI)	Ranking
**Acre**	37.8 (0.0–193.9)	1*	35.2 (2.3–126.0)	1*	27.0 (8.1–67.0)	1*
**Amazonas**	13.2 (7.2–21.4)	2*	11.9 (6.8–18.9)	3*	5.8 (0.9–16.2)	7
**Amapá**	11.4 (0–62.8)	3*	15.9 (0.1–74.9)	2*	14.9 (2.9–37.4)	2*
Maranhão**	9.1 (0.0–53.3)	4	10.5 (1.2–37.4)	6	12.3 (6.7–20.9)	4
**Mato Grosso**	9 (0.0–49.4)	5	10.9 (0.1–47.7)	5	6.5 (1.1–17.6)	6
**Rondônia**	9 (0.0–51.9)	5	10.2 (0.0–46.3)	7	4.9 (0.8–11.8)	8
Roraima**	8.9 (0.0–50.6)	6	11.5 (0.0–51.8)	4	13.3 (1.9–35.5)	3*
**Pará**	8.7(0.0–51.7)	7	10 (0.1–43.9)	8	7.9 (2.9–17.2)	5
**Tocantins**	6.7(0.0–37.3)	8	6.8 (0.1–29)	9	4.4 (1–10.8)	9
**Bahia**	4.5 (0.0–23.1)	9	3.3 (0.0–16.3)	10	2.1 (0.3–5.9)	10
**Ceará**	3.5 (0.0–19.9)	10	3.0 (0.0–13.6)	11	0.9 (0.1–2.3)	11
**Piauí**	2.4 (0.0–14.1)	11	1.3 (0.0–5.7)	14	0.6 (0.2–1.3)	13
**Goiás**	2.3 (0.0–12.2)	12	1.6 (0.0–7.3)	12	0.6 (0.1–1.6)	13
**Alagoas**	2.0 (0.0–10.1)	13	1.4 (0.0–6.5)	13	0.2 (0.0–0.7)	16
**Mato Grosso do Sul**	1.9 (0.0–10.2)	14	1.3 (0.0–5.6)	14	0.7 (0.1–1.9)	12
**Pernambuco**	1.9 (0.0–10.7)	14	1.4 (0.0–6.2)	13	0.4 (0.1–1.0)	15
**Minas Gerais**	1.7 (0.0–9.7)	15	1.6 (0.0–7.0)	12	0.6 (0.1–1.6)	13
**Paraná**	1.6 (0.0–9.2)	16	1.4 (0.0–6.1)	13	0.5 (0.1–1.3)	14
**Espírito Santo**	1.2 (0.0–6.8)	17	1.0 (0.0–4.2)	15	0.4 (0.1–1.0)	15
**Paraíba**	1.2 (0.0–7.1)	17	0.7 (0.0–3.1)	16	0.2 (0.0–0.7)	16
**Distrito Federal**	0.7 (0.0–4.0)	18	0.6 (0.0–2.6)	17	0.2 (0.0–0.6)	16
**Sergipe**	0.7 (0.0–3.7)	18	0.3 (0.0–1.6)	20	0.1 (0.0–0.3)	17
**Rio Grande do Norte**	0.7 (0.0–4.1)	18	0.4 (0.0–1.9)	19	0.1 (0.0–0.5)	17
**Santa Catarina**	0.6 (0.0–3.4)	19	0.5 (0.0–2.3)	18	0.1 (0.0–0.2)	17
**São Paulo**	0.5 (0.0–2.9)	20	0.4 (0.0–1.8)	19	0.6 (0.1–1.6)	17
**Rio de Janeiro**	0.4 (0.0–2.3)	21	0.3 (0.0–1.4)	20	0.1 (0.0–0.2)	17
**Rio Grande do Sul**	0.2 (0.0–0.9)	22	0.1 (0.0–0.4)	21	0.0 (0.0–0.1)	18

YLD: years lived with disability; 95%UI: 95% uncertainty interval;

* The three highest values of YLD according to the ranking of states per year;

** States with increase in YLD values between 1990 and 2000 and 2000 to 2016 comparisons, according to percentage change calculation. CML mortality was assumed to be zero.

## Discussion

To the best of our knowledge, the present study is the first comprehensive assessment of the burden of VL and CML in Brazil and its 27 federated units. The main findings were the considerable changes observed in leishmaniasis burden from 1990 to 2016, with an increase of 83.6% in the age-standardized DALY rate. The increase of leishmaniasis DALY rates was mainly due to VL, which showed growth in all metrics. On the other hand, CML showed a decrease in all metrics, with a decrease of 31.8% of the DALY in the same period. Noteworthy, YLL is the major contributor to the DALY for VL due to the high mortality rates observed for this disease. On the other hand, the cutaneous and mucocutaneous forms usually cause disability rather than death and, therefore, have YLD as the main contributor to the DALY values.

These metrics combine information on mortality and morbidity and allow the estimation of the impact of each disease or injury on the health status of the population, thus constituting a fundamental tool for the elaboration of policies aimed at reducing the burden of diseases [23,24]. In the case of NTDs, the burden imposed by the diseases cannot be calculated considering mortality only, as they usually cause disability rather than leading to death [25,26,27], as in the case of CML. Although some cases of death for CML have been recorded in the SIM database over the years, the GBD study assumes zero mortality for this disease [19]. In 2016, the group of "NTDs and malaria" ranked ninth in the ranking of incidence among communicable, non-communicable diseases, and injuries in Brazil, with an incidence rate of 5,102.6 per 100,000 inhabitants (95%UI 4,228.6–6,181.7). In the same year, the group ranked 18^th^ in the ranking of DALY, with a rate of 256.9 (95%UI 205.6–325.9) per 100,000 inhabitants. VL contributed with 8.1% (20.9/100,000 inhabitants; 95%UI 11.8–34.8) to the DALY rate, while CML contributed with 0.5% (1.5/100,000 inhabitants; 95%UI 0.9–2.3) for the DALY of the same group [28].

In South America, the three countries with the highest DALY values per 100,000 inhabitants for CML in 2016 were: Bolivia (DALY: 60.6; 95%UI 34.7–97.7), Suriname (DALY: 4.8; 95%UI 2.9–7.3), and Peru (DALY: 2.7; 95%UI 1.4–4.7). Brazil is in the fifth position with a DALY of 1.5 (95%UI 0.9–2.3) [28]. In the American countries, of the 46,082 CML cases reported in 2015, 4.2% were mucosal/mucocutaneous. This is considered the most severe CML form as it can lead to clinical complications, disabilities, and mutilations if not diagnosed and treated early [6].

Regarding VL, the ranking of South American countries in 2016 was as follows: Paraguay is the first, with a DALY of 28.5 per 100,000 inhabitants (IU95% 16.2–47.7), followed by Brazil with a DALY of 20.9 (95%UI 11.8–34.87) and Venezuela with a DALY of 0.3 (95%UI 0.1–0.4) [28].

The position of Brazil among the highest rates of DALY for VL indicates a disturbing situation given the severity of this disease. Indeed, YLL has increased 108% between 1990 and 2016. The treatment for VL provided by the Brazilian Public Health System consists of three drug options: pentavalent antimonial (which is considered the first choice), amphotericin B deoxycholate, and liposomal amphotericin B [29,30]. The medication must be taken following medical advice due to its toxicity and common adverse events [29,30,31,32,33]. Although more specific guidelines for the management of patients suffering from severe VL have been developed in Brazil [30], the case fatality rate remains around 8%, which is considered high [34,35].

We observed that all metrics for VL increased over the years, with the incidence rate increasing by 52.9% from1990 to 2016. This may be explained by the geographic changes in the incidence of VL cases which have been occurring in the country since 1980, characterized by the expansion to large urban centers and areas previously free of the disease [36,37,38,39,40].

In the assessment of disease burden for VL, we detected the highest YLL values in children under the age of five, with a higher rate among children under one year of age. In the year 2016 alone, we detected approximately 420 YLLs/100,000 inhabitants in children under 1-year of age, regardless of the gender. This pattern of YLL in children confirms the premature mortality due to VL previously observed in Brazil [35,41,42].

Mortality is practically non-existent for CML. CML mortality was assumed to be null by GBD study, however, the incapacity generated by the lesions in 2016 (YLD) corresponded to an average of one to two YLL per 100,000 inhabitants.

The first-line treatment for CML consists of pentavalent antimony administration for 3–4 weeks and is indicated for the treatment of all forms of tegumentary leishmaniasis. The potential hepato, cardio, and nephrotoxicity of the drugs currently available, associated with their exclusive parenteral administration, represents a great challenge in the search for an adequate and accessible treatment [43,44]. Moreover, the mucous form requires greater care and may present slower responses and higher chances of relapses [44]. It is important to emphasize that most of the cases occur in areas of difficult access, particularly in the middle of the forest in the North region of the country, which makes both the parenteral application of the drug and the monitoring of its side effects difficult [45].

The burdens for VL and CML were higher among males in comparison with females in Brazil. The observed findings may indicate that the risk of infection is often related to occupational and behavioral factors, as job positions predominantly taken by men may propitiate contact with the vector and females are usually more likely to seek health services [30,44,46]. The increased number of CML cases is also linked to environmental changes, such as deforestation, construction of dams, new irrigation schemes, urbanization, and migration of people from non-endemic to endemic areas [2].

Among the Brazilian states, Maranhão (Northeast region) presented the highest values of YLL for VL in 1990 and 2000, followed by the state of Tocantins (North region), which emerged as first in the ranking for this indicator in 2016. In the 1950s, VL was endemic in rural areas in the Northeast region of the country [47]. Later it advanced to other states, reaching the periphery of large urban centers in the states of Pará, Tocantins (North region), Mato Grosso do Sul (Mid-West region), Minas Gerais, and São Paulo (Southeast region) [32,37,38,39,48,49]. Poverty, the presence of migrants in the urban peripheries, and the migratory flow due to drought in certain regions are pointed as some of the factors that explain the high values of VL burden indicators observed in the state of Maranhão in the years of the study [50,51]. For the state of Tocantins, the ecological and epidemiological changes caused by the construction of the capital city, Palmas, led to the urbanization and spread of the disease over the years [52].

The state of Acre (North region) maintained the highest rates of YLD for CML, followed by the states of Amapá, Roraima (North region), and Maranhão (Northeast region). Previous studies have suggested that the expansion of tegumentary leishmaniasis in Brazil is associated with migratory movements originating in the Amazon region. Dispersion of the diseases may have occurred following the return of the population to their regions of origin in the South and Southeast Brazil [53, 54]. The return occurred in corridors with the population leaving the North, passing through the Mid-West and finally reaching the South region of the country [54]. Since then, there has been an increase both in the magnitude and in the geographical expansion of CML cases, with notifications in all regions of the country [34,43,44,45].

The Pan American Health Organization (PAHO) reinforces that VL and CML are NTDs that need continuous efforts for prevention, control, and reduction of incidence in the coming years [55]. Control strategies should include the diagnosis and treatment, integrated vector control, and community health education [56]. In 2016, the Member States of the Pan American Health Organization, under the Resolution CD55.R09 of the Directing Council, approved the regional plan of action for the elimination of neglected infectious diseases and the post-elimination actions for 2016–2022, and defined specific objectives to strengthen the surveillance and control of leishmaniasis in the Americas [6]. With the same purpose, the Leishmaniasis Plan of Action for the Americas 2017–2022 was elaborated detailing the goals, indicators, and lines of action to reduce morbidity and mortality by leishmaniasis in the region [57]. This was also the purpose of the United Nations when launching the 2030 agenda for sustainable development with the goal of drastically reducing the epidemics of AIDS, tuberculosis, malaria and NTDs, among other communicable diseases [58].

The Brazilian Program of Surveillance and Control of Tegumentary Leishmaniasis aims to reduce morbidity, deformities, and deaths. Therefore, diagnosis and treatment are the main strategies of the control program. Likewise, the purpose of the Brazilian VL Surveillance and Control Program (VLSCP) is to reduce the case fatality rate, the degree of morbidity, and the risk of VL transmission. The strategies to achieve these goals include canine serological analysis and euthanasia of infected dogs, vector chemical control, early diagnosis and treatment of human cases, and population awareness [30]. Nevertheless, despite being currently implemented in endemic areas of Brazil, the control interventions for VL remain unsuccessful, and transmission continues, especially in urban areas [59].

The data shown in the present study has limitations with regards to the coverage and quality of the databases used by the GBD and the inequalities among the Brazilian federated units. In addition, it must be taken into account that the cutaneous and mucocutaneous forms present different clinical management. In routine, cutaneous leishmaniasis patients require lower doses of medication, and usually have a good response to the treatment, which is usually not as long as the mucocutaneous form of the disease. In the mucocutaneous form, it would be more prevalent in men, aged over 40 years, with a reduced immune response, and is aggressive, disfiguring and relapsing. Therefore, tegumentary leishmaniasis represents three distinct clinical forms: cutaneous, mucosal and mucocutaneous [8,22,43]. For the next estimates generated in relation to the tegumentar forms, the GBD study should consider them separately due to the differentiated spectrum of the disease.

Indeed, some states, and particularly those located in the North and Northeast regions, still need better coverage and more extensive recording of cases. Nevertheless, the Brazilian health databases have seen considerable improvements in the last decades. The SINAN covers all public and private healthcare systems and their various levels of complexity. Furthermore, the diagnosis and treatment of leishmaniasis are available free of charge in the Brazilian Unified Health System (SUS) [7,8], which has minimized under reporting. Another point to note is that the mortality for CML is assumed to be null by the GBD [20], when in fact it occurs in small numbers in Brazil [8]. From 2007 to 2014, there were 996 deaths due to tegumentar leishmaniasis in Brazil (annual mean of 124.5 deaths), with a cumulative total lethality of 0.55% (0.09% for cutaneous leishmaniasis and 0.46 for other causes) [60]. From this deaths, 155 (15.56%) were due to tegumentar leishmaniasis and 841 (84.44%) were due for other causes [60]. The literature indicates that this low mortality may be related to adverse effects during the treatment of the patients [44,61,62]. Therefore, future estimates should take into account the CML mortality recorded in the country. Nevertheless, the GBD is the most comprehensive and detailed analysis of the burden of leishmaniasis in Brazil and its federated units and represents a major improvement in the evidence base for one of the most neglected tropical diseases.

Assessment of the burden of leishmaniasis helps to understand the dynamics of the diseases and their direct impact on the health status of the population. The data presented here will allow a more accurate interpretation of the published estimates of the burden of leishmaniasis, and the observed regional variations reinforce the need to implement policies adapted to the reality of each region. The regional trends of life years lost due to death and incapacity (DALY) caused by leishmaniasis should be carefully analyzed with the purpose of adopting control strategies specific to the realities of the different Brazilian federated units.

### Conclusions

In summary, the findings of the present study show that the burden of VL increased and of CML decreased over the years in Brazil. The metrics estimated by the GBD 2016 allowed for a better understanding of the burden of VL and CML in the country and its federated units. It was observed that the highest values of YLL for VL are concentrated in the Northeast and Southeast regions due to the urbanization process of the disease. The highest values of YLD for CML in the North region were due to the ecological, social and migratory conditions that favor the occurrence of these forms of leishmaniasis, in the country. The increase of VL cases over the years highlights the need for constant evaluation of the measures of prevention and control of the disease. Furthermore, our analyses provide a contribution to current health policies and might help to reduce the burden and control the disease in Brazil, thus promoting improvements to human and animal health.
